# Long-term plasticity in identified hippocampal GABAergic interneurons in the CA1 area in vivo

**DOI:** 10.1007/s00429-016-1309-7

**Published:** 2016-10-25

**Authors:** Petrina Yau-Pok Lau, Linda Katona, Peter Saghy, Kathryn Newton, Peter Somogyi, Karri P. Lamsa

**Affiliations:** 1 Department of Pharmacology, University of Oxford, Oxford, OX1 3QT UK; 2 MRC Brain Network Dynamics Unit, Department of Pharmacology, University of Oxford, Oxford, OX1 3TH UK; 3 Department of Anatomy, Physiology and Neuroscience, University of Szeged, Közép fasor, Szeged, 6720 Hungary

**Keywords:** LTP, LTD, Parvalbumin, Ivy cell, Interneuron, Oscillation

## Abstract

Long-term plasticity is well documented in synapses between glutamatergic principal cells in the cortex both in vitro and in vivo. Long-term potentiation (LTP) and -depression (LTD) have also been reported in glutamatergic connections to hippocampal GABAergic interneurons expressing parvalbumin (PV+) or nitric oxide synthase (NOS+) in brain slices, but plasticity in these cells has not been tested in vivo. We investigated synaptically-evoked suprathreshold excitation of identified hippocampal neurons in the CA1 area of urethane-anaesthetized rats. Neurons were recorded extracellularly with glass microelectrodes, and labelled with neurobiotin for anatomical analyses. Single-shock electrical stimulation of afferents from the contralateral CA1 elicited postsynaptic action potentials with monosynaptic features showing short delay (9.95 ± 0.41 ms) and small jitter in 13 neurons through the commissural pathway. Theta-burst stimulation (TBS) generated LTP of the synaptically-evoked spike probability in pyramidal cells, and in a bistratified cell and two unidentified fast-spiking interneurons. On the contrary, PV+ basket cells and NOS+ ivy cells exhibited either LTD or LTP. An identified axo-axonic cell failed to show long-term change in its response to stimulation. Discharge of the cells did not explain whether LTP or LTD was generated. For the fast-spiking interneurons, as a group, no correlation was found between plasticity and local field potential oscillations (1–3 or 3–6 Hz components) recorded immediately prior to TBS. The results demonstrate activity-induced long-term plasticity in synaptic excitation of hippocampal PV+ and NOS+ interneurons in vivo. Physiological and pathological activity patterns in vivo may generate similar plasticity in these interneurons.

## Introduction

Activity-induced long-term plasticity characterizes neuronal communication widely in the brain providing cellular level mechanisms for learning and memory (Morris 2013). Various long-term plasticity forms have been characterized in interactions between glutamatergic neurons in the cortex in ex vivo slice preparation and in the intact brain in vivo. These include activity-induced synaptic long-term potentiation (LTP) and -depression (LTD), and changes in postsynaptic neuron excitability (Collingridge et al. 2010; Luscher et al. 2000; Lisman and Spruston 2005; Daoudal and Debanne 2003). Studies in ex vivo slice preparation have reported long-term plasticity also in glutamatergic excitation of many cortical GABAergic interneurons (Kullmann et al. 2012; Kullmann and Lamsa 2011; Topolnik 2012; Laezza and Dingledine 2011; Bartos et al. 2011; Galvan et al. 2011; McBain 2008). Experiments in hippocampal slices have demonstrated LTP in interneurons in CA1 that requires NMDA receptors (Lamsa et al. 2005, 2007a; Le Roux et al. 2013) as well as different forms of long-term potentiation independent of NMDARs (Perez et al. 2001; Le Duigou and Kullmann 2011; Pelkey et al. 2008; Topolnik et al. 2006; Camire and Topolnik 2014; Lamsa et al. 2007b; Galvan et al. 2008; Nicholson and Kullmann 2014; Hainmuller et al. 2014; Campanac et al. 2013). In addition, excitatory synapses in CA1 interneurons exhibit long-term depression by endocannabinoids (Peterfi et al. 2012; Edwards et al. 2012). Although only few studies have investigated long-term plasticity in interneurons in vivo, potentiation and depression akin to LTP and LTD between some CA1 interneurons and their afferent excitatory fibers have been reported after high-frequency electrical stimulation or by learning-driven hippocampal activity (Buzsaki and Eidelberg 1982b; Dupret et al. 2013). However, the hippocampal CA1 area contains a great diversity of GABAergic interneuron types with specialized activity and connectivity (Somogyi and Klausberger 2005). Whether the different types of identified interneurons show plasticity in vivo and how this plasticity is regulated by brain states are unknown. Importantly, ex vivo studies have shown that long-term plasticity in CA1 interneurons is regulated by neuromodulator mechanisms from extrahippocampal sources such as cholinergic transmission (Le Duigou et al. 2015; Griguoli et al. 2013). The activity of many of these ascending fibers is brain state dependent (Vandecasteele et al. 2014) suggesting that interneuron plasticity might also differ during different brain states.

In the present study, we have investigated activity-induced long-term plasticity in synaptically evoked firing of identified CA1 interneurons in vivo in rats under urethane anesthesia in order to improve recording stability, and compared plasticity results after TBS stimulation during different oscillatory network states recorded as local field potential.

## Methods

### Animals and surgical procedures

Experiments were carried out on adult male (weight 280–350 g) Sprague–Dawley rats (Charles River, UK) according to the Animal Scientific Procedures Act, 1986 (UK) using a heating mattress (37.5 ± 0.5 °C) with an external abdominal temperature measurement probe with feedback to the heating pad. Anesthesia was induced with isoflurane (4 % v/v in O_2_) and maintained by a single intraperitoneal (i.p.) injection of urethane (1.25–1.3 mg/kg in 0.9 % saline, i.p.). Ketamine (30 mg/kg i.p.) and xylazine (3 mg/kg i.p.) were given at the start of the procedure and in supplementary small doses during recording to maintain anesthesia. Saline-based glucose solution (5 % v/v glucose) was injected subcutaneously (2 ml/2 h) to compensate for fluid loss during the experiment. A rostrocaudal incision was performed to expose the skull, and surgical windows were made above the right and left dorsal hippocampal CA1 areas with a dental drill. A wall of dental cement was built to protect the openings and saline was applied regularly to the exposed brain surface. For accurate measurement of penetration depth, saline solution was drained before inserting electrodes into the brain. The windows were covered with warm paraffin wax once the electrodes were lowered into the brain.

### Electrophysiological recording, cell labeling, data acquisition and electrical stimulation

Microelectrodes were pulled from borosilicate glass capillaries (GC120F-10, Harvard Apparatus, UK) using a vertical puller (PE-2, Narishige, Japan) and were filled with 1.5–3 % (w/v) neurobiotin (Vector Laboratories, UK) in 0.5 M NaCl. The recording electrodes were lowered into the brain at 20 µm/s, and into the hippocampus at 5 µm/s using a micro drive holder (EXFO-8200 IMMS, Canada) and a computer-controlled 0.5 µm-stepping interface. Stereotaxic co-ordinates for the recording electrodes were: 3.0 mm posterior to Bregma (±0.3 mm), 3.6 mm from midline (±0.5 mm), and depth 2.2 mm (±0.3 mm). The electrode resistance was 15–21 MΩ.

Following extracellular recording, the electrode was moved into juxtacellular position and the recorded cells were modulated by applying a series of +10 to +50 nA square pulses of 200 ms duration in 30 s episodes for 2–3 minutes continuously (Brown et al. 2009). We verified that the action potential properties (extracellular spike kinetics) of the modulated cell corresponded to the action potential properties recorded during plasticity experiment. This labeling procedure was followed by a period from 1 to 5 hours (Klausberger et al. 2005), which allowed for the diffusion of neurobiotin inside the modulated cells.

Signal was amplified 1000× (10×, head-stage amplifier, Axon Instruments, USA; 100×, NL-106, DigitimerTM, UK) and band-pass filtered between 0.3 and 300 Hz for local field potentials (LFP) and between 300 Hz and 5 kHz for detection of single spikes. The LFP and single neuron activity were acquired at 1 and 19.841 kHz, respectively using Spike2 (version 7.0; Cambridge Electronic Design, UK).

Concentric bipolar stimulating electrodes (125 µm tip diameter, FHC Inc., USA) were stereotaxically placed in the left hippocampal CA1 area 3.0–3.2 mm posterior and 3.0–4.0 mm lateral to Bregma and at 2.1–2.5 mm depth from the cortical surface (Buzsaki and Eidelberg 1982b). Single-shock stimulation (100 µs, 150–600  µA) was delivered every 5 s using current isolator stimulator (DS3; Digitimer, UK) to elicit spikes. The train of theta-burst stimulation (TBS) consisted of 20 bursts (at 200 ms intervals) of five stimuli at 100 Hz.

### Data analysis and statistics

Data analyses were performed using Spike2 and MATLAB (MathWorks). The spike shape, width and amplitude were carefully monitored throughout the experiment and compared between spontaneous and evoked spikes using Principal Component Analysis (PCA) in Spike2. In the LFP, periods of theta frequency (3–6 Hz) oscillation were identified off-line using custom-made script in Spike2 (Tukker et al. 2007) as at least three consecutive windows of 2 s each during which the ratio between the power in 3–6 and 2–3 Hz frequency bands was >4. The start and end point of theta cycles defined by the script were confirmed by visual inspection. Theta oscillatory cycle troughs were identified and the theta phase of the single neuron action potentials was established (Tukker et al. 2007). The spontaneous firing was considered modulated by theta oscillations when the phases of action potentials were non-uniformly distributed along theta cycles (*P* ≤ 0.05, Rayleigh’s method). Phase histograms with 18° bin size were used to illustrate the average phase coupling to theta cycles. The preferential mean angle of firing was calculated using normalized vector addition (Klausberger et al. 2005).

To test for any changes in the LFP caused by TBS, we have calculated the wavelet power spectrogram of the LFP in 2 s time windows before and after TBS. The LFP signal was wavelet transformed using complex Morlet function (nondimensional central frequency of 6) evenly spaced between 1.25 and 40 Hz on a log scale. From this, the power spectrogram was calculated as the wavelet amplitude squared. We have derived an LFP index by calculating the average wavelet power in the frequency bands 1–3 Hz (avgPower1_3Hz) and 3–6 Hz (avgPower3_6Hz), respectively, restricted to 1 s before and after TBS and using the formula,

$$ \frac{{{\text{avgPower}}3\_6{\text{Hz }}{-}   \,{\text{avgPower}}1\_3{\text{Hz}}}}{{{\text{avgPower}}3\_6{\text{Hz }} + {\text{avgPower}}1\_3{\text{Hz}}}}, $$

where index values of 1 and −1 represent spectral power components only in the frequency ranges 3–6 or 1–3 Hz, respectively; whereas 0, represents exactly the same average power in both frequency ranges. The average wavelet power across a range of frequencies (e.g. 1–3 and 3–6 Hz) was determined as the weighted sum of the wavelet power spectrum over the respective frequencies.

Stimulus-evoked fEPSP and spikes were analyzed using Spike2. fEPSP onset and time to peak values as well as initial slope (from onset to 30 %) were determined from 3 kHz off-line low-pass filtered raw signals. Evoked spikes were detected from band-pass filtered 300 Hz–5 kHz signals. In each cell, episodes of consecutive (at least 120) “monosynaptic time windows” (up to 15 ms) following stimulation were compared with episodes of spontaneous activity in similar (15 ms) time windows immediately before the stimulation to test whether the number of spikes was different in the two periods (Chi-square test) (Buzsaki and Eidelberg 1982a, b). During baseline conditions, i.e., before TBS, the 13 of 72 cells reported here showed higher number of cases with spikes in the “monosynaptic time window” than in the equal time window preceding the stimulation (Chi-square test). None of the seventy-two cells showed significantly higher number of time windows with spikes in the late period (post-stimulation 15–50 ms) compared to periods preceding the stimulation (measured in 35–0 ms before stimulation) in at least 120 consecutive windows. However, six cells fired with lower rates during the 15–50 ms post-stimulation period (at least 120 for each cell) than in 35–0 ms before stimulation (for each cell *P* < 0.05, Chi-square test). Changes in evoked spike probability (as failure or spike in 3–15 ms from stimulation, at least 120 windows) before and after TBS (post-TBS) in individual cells was analyzed using Chi-square test. ANOVA and post hoc Bonferroni test for multiple comparisons or *t-*test were used to analyze changes in spike delay. Using *t-*test the evoked spike probability, spike delay, or spike delay time 1/CV^2^ values were compared between baseline and post-TBS in groups of cells. Spikes during TBS were summed between the 1st stimulation and 50 ms after the 5th stimulation of each burst. *P-*values <0.05 indicate significant difference in the mean firing rates between post-TBS periods and baseline.

Spike probability and spike delay time values (including the 1/CV^2^) were normalized using the baseline average for presentation and analyses in Fig. 6 to show a relative change.

Spontaneous firing levels were calculated during 1 s episodes immediately preceding the stimulations throughout the experiment. For presentation in figures and for statistical analyses, values of spontaneous firing level (number of spikes in 1 s windows) data were pooled giving average number of spikes in 12 consecutive episodes of 1 s, every 5 s resulting in 1 min bin. Statistical comparison of spontaneous firing level during baseline and post-TBS periods was performed using ANOVA and Bonferroni test for multiple comparisons and comprised at least ten consecutive 1 min bins for each condition.

Normal distributions of data were tested with Kolmogorov–Smirnov test. Data are shown as mean (±SEM) unless otherwise stated. For testing correlation and significance in scatter plots of data in Fig. 6 data have been fitted to the coefficient of determination (*r* ^2^) and tested with the Pearson’s test.

### Visualization of recorded neurons, immunohistochemistry and electron microscopy

Animals were perfused with 0.1 M phosphate buffered saline solution (PBS, pH 7.4, at 22–24 °C) followed by ice-cold fixative solution; 4 % w/v paraformaldehyde (PFA) with 15 % v/v saturated picric acid solution in 0.1 M phosphate buffer (PB) with freshly added glutaraldehyde to a final content of 0.05 % w/v. Brains were kept in fixative (4 % PFA in 0.1 M PB) at 4 °C for 24 hours, then stored in 0.1 M PB with 0.05 % sodium azide preservative at 4 °C. Vibratome (VT1000S Leica Microsystems, UK) was used for cutting coronal brain sections (60–70 µm thickness). Sections were washed in PB three times for 10 minutes at 24.0 °C, then incubated overnight in streptavidin-conjugated AlexaFluor488 in 0.1 M Tris-buffered saline (TBS, pH 7.4) with 0.3 % TritonX-100 (Sigma-Aldrich Inc., USA) on a shaker at 4 °C. Sections were mounted using Vectashield (Vector Laboratories Inc, USA) under cover slips and were examined using epifluorescence microscopy (AxioImager Z1, Carl Zeiss, UK). Some sections used for electron microscopy were cryoprotected in 20 % sucrose dissolved in PB and permeabilized using a ‘freeze–thaw’ procedure instead of Triton-X treatment.

For immunohistological reactions, free-floating sections were washed three times in TBS (15 minutes) at 24 °C, and then incubated in 20 % horse or goat serum in TBS for blocking non-specific antibody attachment. The sections were incubated in primary antibodies diluted in 1 % horse or goat serum in TBS over two nights at 4 °C. After washes, the same sections were incubated in relevant fluorochrome-conjugated secondary antibodies in 1 % of blocking serum in TBS for overnight at 4 °C, or 2–4 hours at 24.0 °C. Sections were washed in TBS (20 minutes) three times, and mounted on glass slides. The characterizations of antibodies (host, dilution, source) to cannabinoid receptor CB1 (goat, 1:1000, Frontier Institute Co. Ltd., Hokkaido, Japan, http://www.frontier-institute.com), neuronal nitric oxide synthase (mouse, 1:2000, Sigma, USA), neuropeptide Y (NPY, rabbit, 1:5000, ImmunoStar, Inc., USA, http://www.immunostar.com), parvalbumin (rabbit, 1:1000, Swant, Switzerland, http://www.swant.com), pro-CCK (rabbit, 1:500, Frontier Institute Co. Ltd., Japan), special AT-rich sequence-binding protein-1 (SATB1, goat, 1:1000, Santa Cruz Biotechnology Inc., USA, http://www.scbt.com.) have been reported earlier (Viney et al. 2013; Unal et al. 2015). Fluorophore-labeled secondary antibodies are described in Unal et al. (2015). Labelling of neurons using neurobiotin and immunoreactions were evaluated using both epifluorescence (Ferraguti et al. 2005) and laser scanning confocal microscopy (Somogyi et al., 2012). Immunoreaction was considered to be negative when fluorescence was not detected in relevant neurobiotin-labelled cell, but immunopositivity was detected in the same area in unlabeled cells.

Following fluorescence microscopic analysis, neurobiotin was revealed by horseradish peroxidase reaction, the sections were treated with osmium tetroxide for increasing contrast, dehydrated and mounted on slides in epoxy resin for light microscopic identification of the axonal and dendritic patterns (Viney et al. 2013). Some cells were partially reconstructed from serial sections using a drawing tube and a PL Apo 63×/1.4 numerical aperture (n.a.) oil immersion objective. The axon terminals of two neurons were evaluated by electron microscopy as described earlier to aid their identification by revealing some of the postsynaptic elements (Viney et al. 2013).

### Confocal microscopy

Parameters and methods used for confocal microscopic image acquisition were as reported earlier (Viney et al. 2013), except that in some cases multiple channels were captured in the same track, if they were suitably distant from each other in wavelength. Briefly, multi-channel fluorescence images were acquired using ZEN 2008 software v5.0 on a Zeiss LSM 710 laser scanning confocal microscope (Zeiss Microscopy GmbH, Germany), equipped with DIC M27 Plan-Apochromat 40×/1.3 n.a., DIC M27 Plan-Apochromat 63×/1.4 n.a. and alpha Plan-Apochromat 100×/1.46 n.a. oil immersion objectives. Channel specifications were (laser/excitation wavelength, beam splitter, emission spectral filter) for detection of Alexa405, 405–430 solid state 405 nm with attenuation filter ND04, MBS-405, 409–499 nm; for Alexa488, argon 488 nm, MBS-488, 493–542 nm; for Cy3, HeNe 543 nm, MBS-458/543, 552–639 nm; For Cy5, HeNe 633 nm, MBS 488/543/633, 637–757 nm. Pinhole sizes were chosen by selecting 1 Airy Unit for the channel of the shortest wavelength, and matching the resulting optical section height across the other channels. For illustrations, all manipulations to brightness and contrast, and median filtering (if applied), were carried out on whole images, not selectively.

## Results

### Short delay synaptic excitation in CA1 area from the contralateral hippocampus

**Fig. 1:**
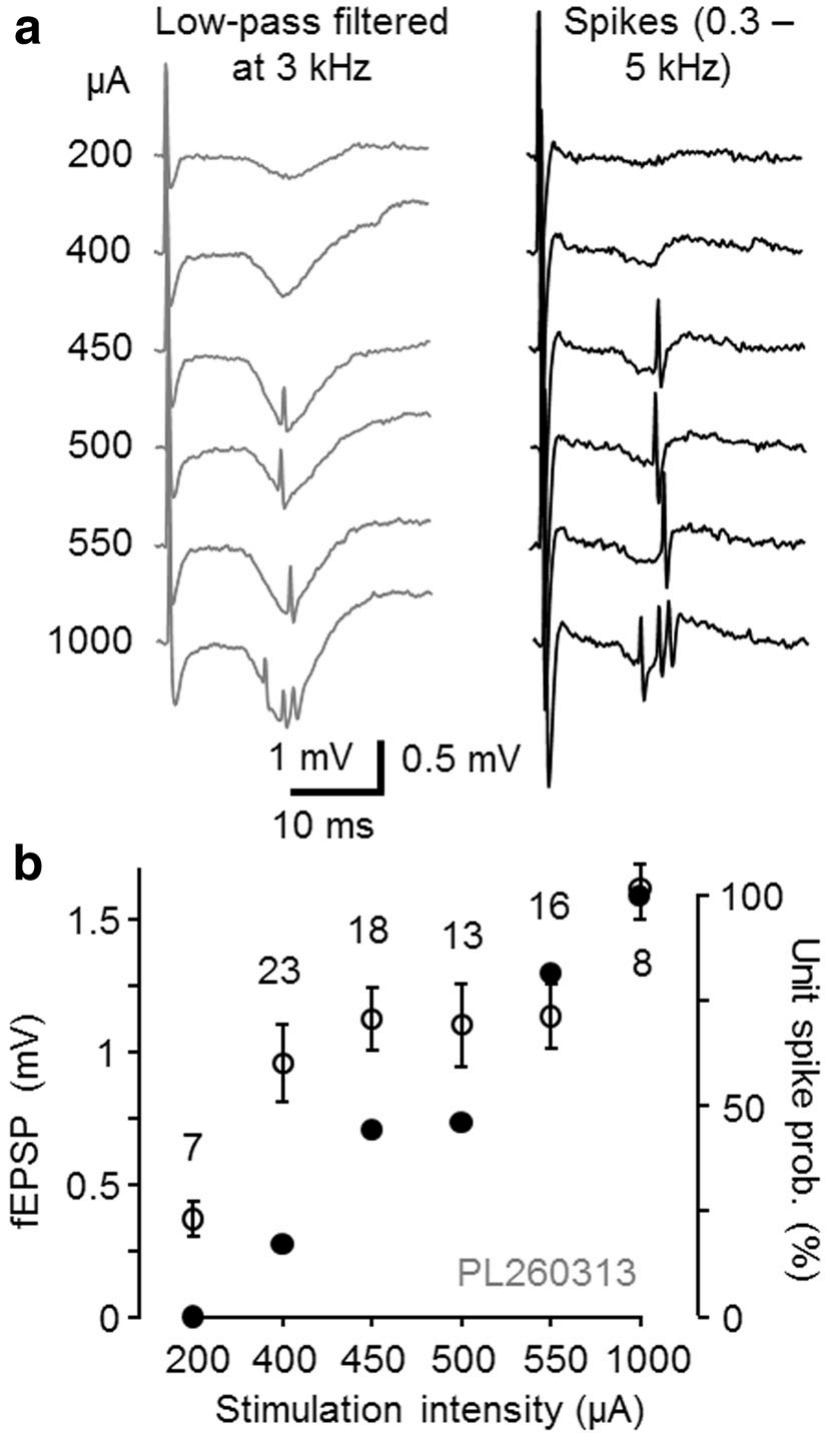
Electrical stimulation from contralateral hippocampus elicits fEPSP and postsynaptic action potential with phase-locked short delay in CA1. **a** Extracellularly recorded traces from one experiment with fEPSP and postsynaptic cell spikes evoked by different stimulus intensities from contralateral CA1 area. Increasing the stimulation intensity (*left* μA) augmented fEPSP amplitude (signals low-pass filtered at 3 kHz) and action potential (spikes, band-pass filtered between 0.3 and 5 kHz) probability without a significant effect on the spike delay indicating monosynaptic transmission. **b** Amplitude of fEPSPs (*open symbols* mean ± SD) and spike probability (*solid symbols*) of a pyramidal cell (PL260313) evoked with different stimulus intensities. Intensity was finally set to elicit spikes with approximate half-maximal probability

Electrical stimulation was applied from the contralateral (left) hippocampal CA1 area aiming to antidromically activate CA3 pyramidal cells and their Schaffer collaterals in the right hemisphere and antidromically stimulate left hippocampal CA3 pyramidal cells and their commissural fibers to the right hippocampal CA1 (Buzsaki and Eidelberg 1982a, b). We first confirmed that single-shock stimulation was able to elicit field EPSP (fEPSP) in the right hippocampal CA1 with a short phase-locked delay (6.23 ± 0.43 ms to the fEPSP onset, *n* = 13) and short time to peak (from onset 2.52 ± 0.63 ms, *n* = 13) (Buzsaki and Eidelberg 1982b; Bliss and Lomo 1973) (Fig. 1a).

Action potentials of individual CA1 area neurons were measured extracellularly (Fig. 1a). In 13 out of 72 spontaneously active cells recorded in the CA1 area, phase-locked action potential to single-shock stimulation (interval 5 s) was elicited with a short delay (9.95 ± 0.41 ms, *n* = 13, see methods for criteria) that corresponds to monosynaptic excitatory pathway (Buzsaki and Eidelberg 1982a, b). The probability of the phase-locked spike was modulated by stimulation strength with minor alterations in spike latency (Fig. 1b). Stimulus intensity was then adjusted to elicit the spike with an approximate half-maximal probability. In 59 out of the 72 cells, single-shock stimulation failed to elicit spikes (see “Methods”). Antidromic action potentials due to stimulation were not observed in the cells reported here (Buzsaki and Eidelberg 1982b).

### Long-term potentiation of synaptic excitation in identified CA1 pyramidal cells

**Fig. 2:**
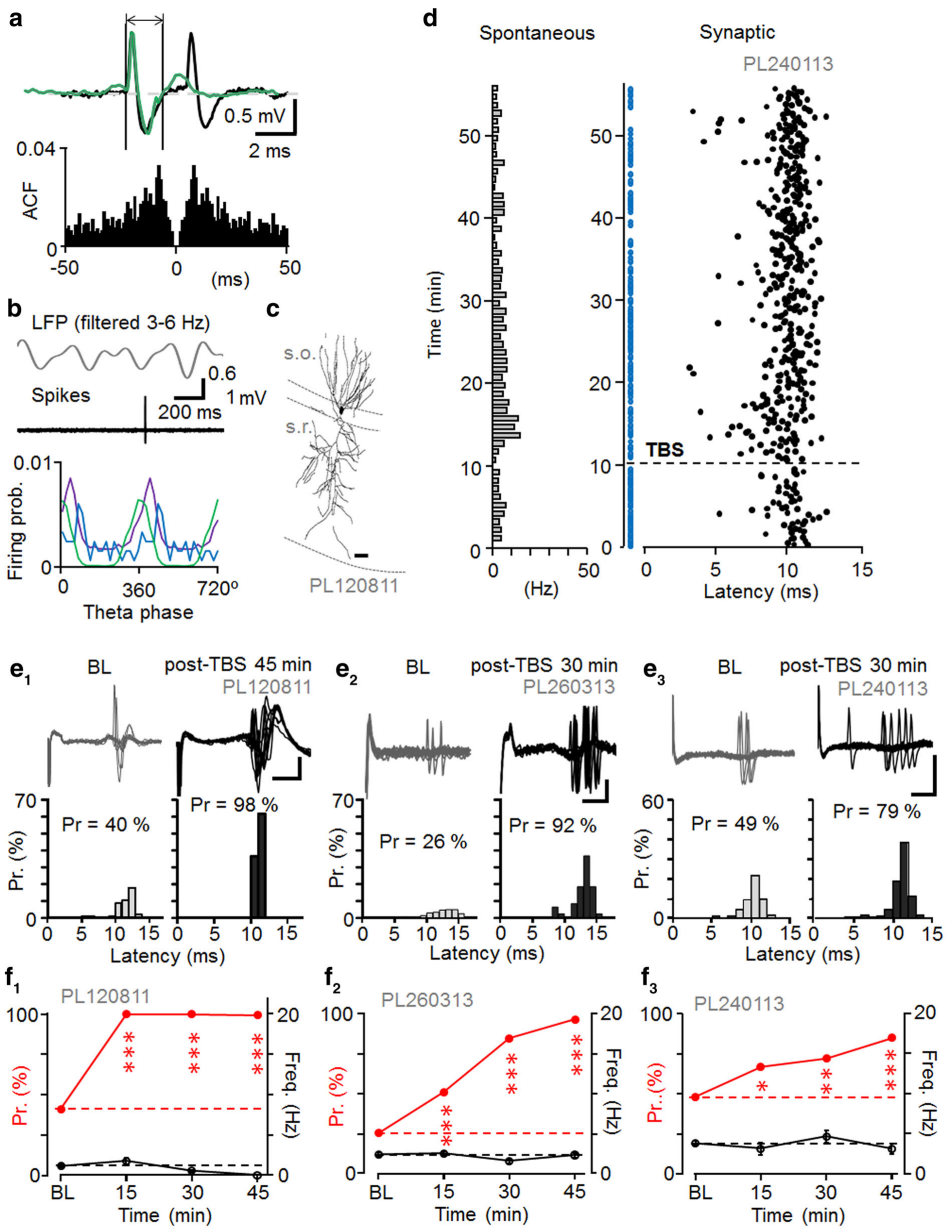
Long-term potentiation of synaptic excitation in identified CA1 pyramidal cells. **a** Occasional complex spikes characterized spontaneous CA1 pyramidal cell firing. *Top* spontaneous single spike (*green*) and complex spike (*black*) superimposed from the same cell. *Vertical lines* define onset and the end of single spike showing slow (>1.5 ms) spike duration. *Bottom* autocorrelogram of spontaneous spiking. A peak at approximately 10 ms reflects the spike interval of complex spikes. **b** Pyramidal cells fired spontaneously with highest probability around the trough of LFP theta cycles. *Top* band-pass filtered LFP (3–6 Hz) and a spontaneous pyramidal cell spike (band-pass filtered at 0.3–5 kHz) during theta oscillations. *Bottom* spike timing histogram showing firing preference of the pyramidal cells around the theta cycle trough (*n* = 3, cells shown in *different colors*). **c** Recorded pyramidal cell partially visualized from one 60 μm thick section. *Scale* 20 μm. **d** Synaptically evoked spike probability and delay (latency) to single-shock stimulation in one pyramidal cell during baseline and after theta-burst stimulation of the contralateral hippocampus (TBS, *horizontal dotted line*). Stimuli failing to evoke spike are shown with *blue dots* (abscissa). Spontaneous firing of the cell is shown in Hz as bar histogram on the left (1 min bin). **e**, **f** Long-term potentiation (LTP) in the synaptically-evoked spike probability in three identified pyramidal cells after the TBS. **e1**–**e3** *Top* superimposed traces showing synaptically-evoked spikes with occasional failures in the three cells during baseline (BL) and after the TBS (post-TBS). *Scales* 1, 0.2, and 0.5 mV, respectively; 5 ms. *Bottom* histograms show increased spike probability (Pr) (Chi square test), but unaltered spike latency post-TBS (*t* test). The potentiation is significant in each cell (*P* < 0.005). **f1**–**f3** Spike probability in the three cells at different time points (*red symbols* scaling left, significance compared to baseline, Chi square test). *Black symbols* show spontaneous firing (scaling right). Spontaneous firing level was significantly reduced long-term from baseline only in **f1** at the last two time points (ANOVA with Bonferroni test)

Three postsynaptic pyramidal cells were identified by their spontaneous electrical activity and spike waveform, and two of them were confirmed by visualization (Pyapali et al. 1998). The pyramidal cells fired spontaneously at low rate with the highest probability close to the LFP theta cycle trough. They showed characteristic slow spike kinetics (Table 1) and two of three cells showed occasional complex spikes (Fig. 2a–c) (Harris et al. 2001).

**Table 1:** Summary of spontaneous firing properties and spike kinetics of pyramidal cells

Spontaneous spiking properties				
Cell code	Firing rate during LFP theta (Hz)	Mean angle phase (±cSD) to LFP theta cycle (°)	Depth of modulation (*r*) during the theta and *n* of cycles	Spike durations (ms) (mean ± SEM)
PL120811	2.94	84 ± 64	0.38, *n* = 34	1.98 ± 0.04
PL240113	3.29	34 ± 49	0.64, *n* = 852	1.90 ± 0.04
PL260313	2.96	358 ± 40	0.76, *n* = 833	1.41 ± 0.04

The three pyramidal cells showed low spontaneous firing rate during theta oscillations in the LFP. The cells fired with highest probability close to LFP theta cycle troughs (180° peak of cycle, cSD means circular standard deviation). Extracellularly recorded action potential showed slow kinetics (close to 1.5 ms or longer)

Pyramidal cells generated spikes to afferent stimulation with 11.44 ± 0.71 ms delay (*n* = 3) at approximate half-maximal spike probability, which in two experiments was associated with fEPSPs. After recording baseline (at least 10 min), high-frequency theta burst stimulation (TBS, 5 pulses at 100 Hz, repeated 20 times at 200 ms interval) was delivered in the left CA1, whereupon the single-pulse stimulation was resumed (Fig. 2d). The TBS increased the stimulus-evoked spike probability for at least 45 min in all three pyramidal cells (*n* = 3, for each cell *P* < 0.005, Chi-square test) while the average spike delay to the stimulation remained unchanged (*n* = 3, *t* test) making it unlikely that the potentiation was conveyed polysynaptically via a recurrent circuit (Buzsaki and Eidelberg 1982b; Maccaferri and McBain 1996). While the spike probability potentiated from 0.38 ± 0.07 during baseline to 0.93 ± 0.04 in 15–45 min during post-TBS (*n* = 3, *P* < 0.005, *t* test) the mean spike delay time remained unchanged (*t* test) in the three cells (post-TBS 11.29 ± 0.79 ms). The coefficient of variance (CV) of the delay time decreased in two of the three cells, but 1/CV^2^ of the three cells was not significantly changed after LTP (15–45 min) (*n* = 3, *t* test). Baseline-normalized 1/CV^2^ for the three cells after LTP (15–45 min) was 2.41 ± 1.18. In the two experiments with fEPSP, the spike probability increase was accompanied by LTP in fEPSP initial slope to 127 and 124 % from baseline, respectively (at 15–45 min post-TBS, for each recording *P* < 0.01, *t* test). Spontaneous firing level remained unchanged between the baseline and the period following TBS in two cells and was reduced after TBS in one pyramidal cell (*P* < 0.01, *t* test) (Fig. 2e, f; Table 2). In two out of three experiments 1–3 Hz oscillations occurred in the local field potential (LFP) at the time of TBS application (measured over 1 s prior to TBS), whereas in one experiment the LFP was dominated by 3–6 Hz oscillatory activity (see Table 7 for summary of all 13 cells in this study). Data on the stimulus-evoked spikes and level of spontaneous firing in the plasticity experiments are summarized in Table 2.

**Table 2:** Summary of pyramidal cell firing

Synaptically evoked spike properties and spontaneous firing rate during plasticity experiment									
Cell code	Probability to the afferent stimulation			Latency to the afferent stimulation (ms) (mean ± SEM)			Overall spontaneous firing of the cell during the experiment (Hz)		
	Baseline	Post-TBS (0–15 min)	Post-TBS (15–45 min)	Baseline	Post-TBS (0–15 min)	Post-TBS (15–45 min)	Baseline	Post-TBS (0–15 min)	Post-TBS 15–45 min)
PL120811	0.41	1.00***	0.99***	11.79 ± 0.11	10.17 ± 0.06	11.06 ± 0.03	1.11	1.78	0.32**
PL240113	0.48	0.64*	0.85***	10.01 ± 0.16	9.64 ± 0.26	10.05 ± 0.23	3.78	3.15	3.12
PL260313	0.26	0.51***	0.96***	12.35 ± 0.48	13.51 ± 0.29	12.78 ± 0.23	2.43	2.12	2.36

Average probability (Chi square test) and latency of synaptically evoked postsynaptic spikes (ANOVA with Bonferroni test) in the experiments during baseline and at two different periods following TBS (post-TBS). The overall spontaneous firing frequency in the baseline and at early (0–15 min) and late (15–45 min) post-TBS time windows are shown at right

* *P* < 0.05, ** *P* < 0.01, *** *P* < 0.005

### Plasticity of synaptic excitation in fast-spiking PV+ interneuron types

**Fig. 3:**
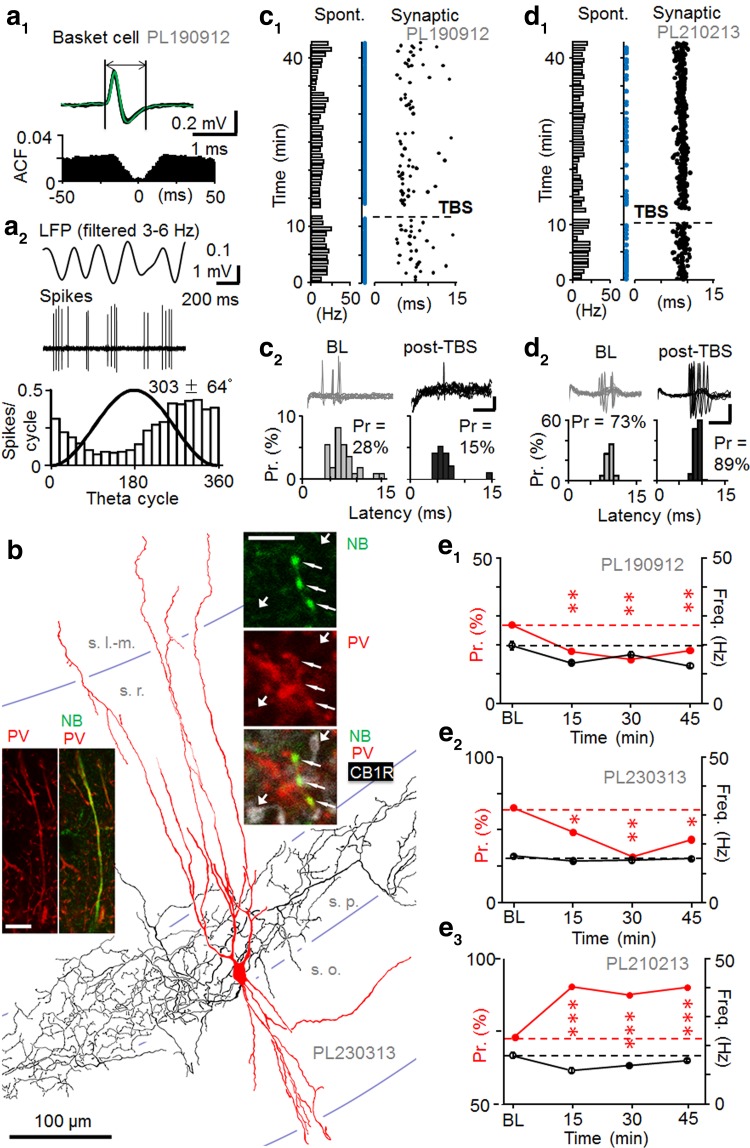
Long-term plasticity of synaptic excitation in PV+ basket cells. **a** Spontaneous firing and spike properties of a PV+ basket cell. **a1** *Top* sample traces showing fast-kinetic action potentials of a basket cell (PL190912). The *vertical lines* define spike duration. *Bottom* autocorrelation of spontaneous firing lacks a clear peak. **a2** Spontaneous firing during theta frequency (3–6 Hz) oscillations. *Top* traces show LFP theta oscillations (band-pass filtered 3–6 Hz) and basket cell spikes (band-pass filtered at 0.3–5 kHz). *Bottom* histogram shows firing preference (18° bins) along the descending slope (mean ± cSD) of the LFP theta cycle (*black line* sine wave). **b** Partial reconstruction of the dendrites (*red* from four 70 µm-thick sections) and axon (*black* from two sections) of a recorded basket cell. Confocal microscopic images show parvalbumin immunopositivity (*red*) in neurobiotin-labeled (NB, *green*) dendrite (*left*, *scale* 10 µm) and boutons (*right*, *thin arrows*, *scale* 5 µm). The boutons were immunonegative for CB1 receptor (*white*, CB1R) evident from neurobiotin-free boutons nearby (*thick arrows*). **c** LTD in an identified basket cell. **c1** Raster plot shows synaptically-evoked spikes (*black dots*) and stimuli with spike failures (*blue dots*). Spontaneous firing shown on *left* (1 min bins). **c2** Synaptically-evoked spikes during baseline and 15–30 min after TBS. *Top* superimposed traces showing spikes at baseline and post-TBS (*scales* 0.2 mV, 5 ms). *Bottom* histograms of spike probability (Pr, %) and delay (latency). Spike probability decreased after TBS (*P* < 0.01, Chi square test). **d** Corresponding data from another basket cell showing long-term potentiation of Pr after TBS (*P* < 0.005, Chi square test). *Scales* 0.2 mV, 5 ms. **e** Synaptic spike probability in three identified basket cells. **e1**–**e3** Tested for plasticity and showing either LTD-like decreased Pr or LTP-like increased Pr. Post-TBS time points are compared to baseline (Chi square test). Spontaneous firing level was significantly reduced long-term (>15 min post-TBS) from baseline in cell PL190912 (ANOVA with Bonferroni test)

**Fig. 4:**
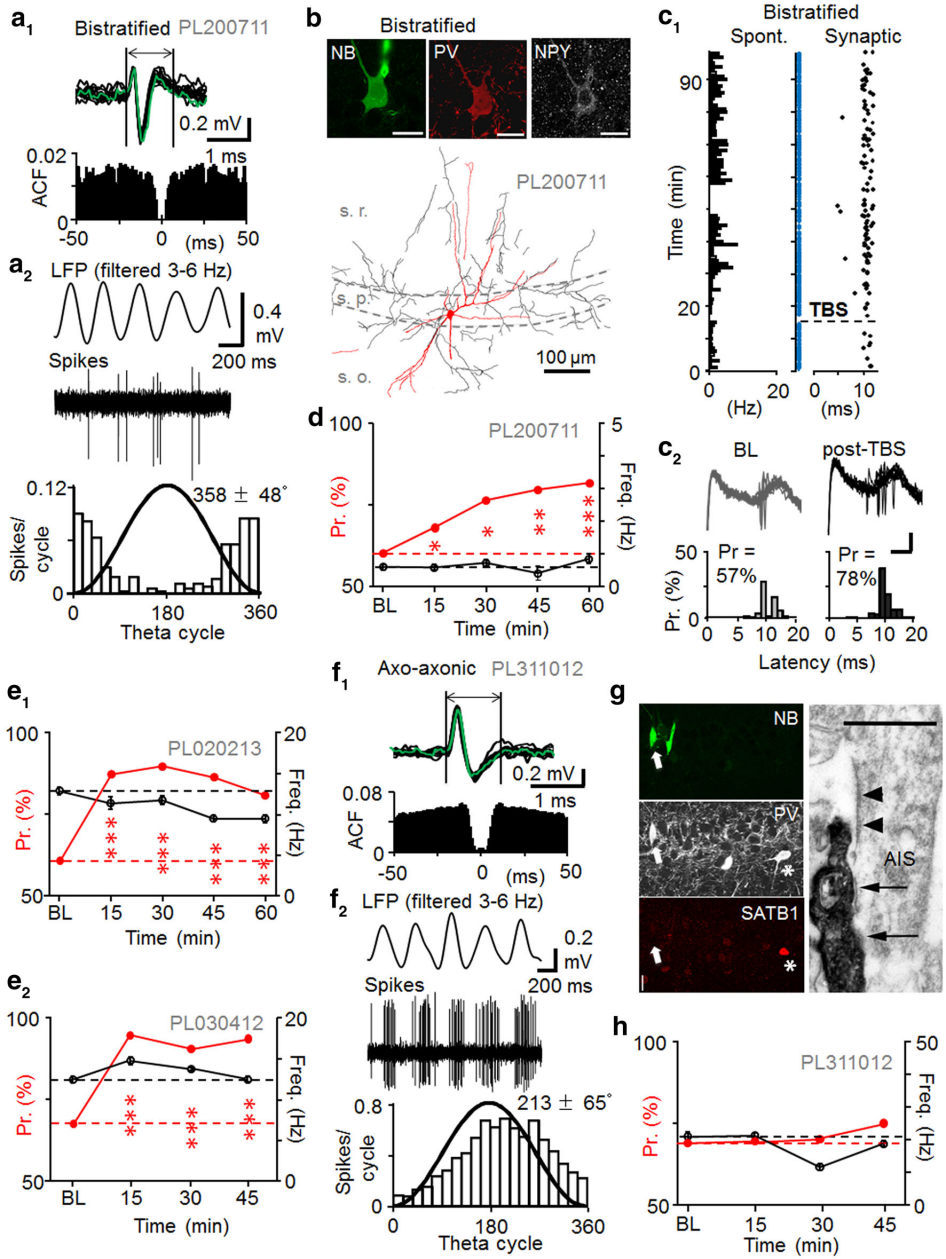
Long-term plasticity in fast-spiking interneurons. A bistratified cell and two unidentified fast-spiking interneurons. **a** Spontaneous firing of a bistratifed cell (PL200711). **a1** *Top* fast-kinetic action potentials of the cell. *Bottom* autocorrelation lacks a distinct peak within 50 ms. **a2** Spontaneous firing of the cell during theta frequency (3–6 Hz) oscillations. *Top* band-pass filtered LFP (3–6 Hz) and bistratified cell spikes (band-pass filtered at 0.3–5 kHz). *Bottom* spike occurrence histogram shows firing preference (mean ± cSD) at the trough of LFP theta cycle (18° bins; *black line* sine wave). **b** The bistratified cell soma and proximal dendrites are immunopositive for PV (Cy3) and NPY (Cy5) as shown by confocal microscopy (*top*). *Scale* 20 μm. *Bottom* reconstructed soma and dendrites (*red*), and axon (*black*) of the cell in one 70 μm-thick section. Note distribution of the axon in *strata radiatum* (s.r.) and *oriens* (s.o.). **c** Synaptically evoked spike probability in the bistratified cell showed persistent potentiation after TBS. **c1** Raster plot of synaptically-evoked spikes (*black dots*) before and following TBS (*dotted horizontal line*) in the cell. Spike failures to stimuli are shown in *blue*. Spontaneous firing of the cell shown on the *left* (1 min bins). **c2** Superimposed LFP traces with spikes and histograms show probability (Pr) and delay (latency) in baseline and 45–60 min after the TBS. *Scales* 0.2 mV, 5 ms. **d** Average synaptically-evoked spike probability in the cell at different time points (Chi square test). Spontaneous firing level was altered from baseline at 45 min and 60 min post-TBS time (*P* < 0.05, ANOVA with Bonferroni test). **e** Two fast-spiking unidentified interneurons show persistent potentiation of Pr after TBS (Chi square test). **e1** LTP in PL020213 was associated with decreased spontaneous spiking at late post-TBS times (45–60 min, *P* < 0.05, ANOVA with Bonferroni test). **e2** The LTP in PL030412 was associated with transiently increased spontaneous firing level at 15 min post-TBS time (*P* < 0.05, ANOVA, with Bonferroni test). **f**–**h** Lack of lasting plasticity of synaptically-evoked spike probability in an identified axo-axonic cell. **f** Spontaneous firing and spike properties of the axo-axonic cell. **f1** *Top* traces show fast-kinetics of the action potential. *Bottom* autocorrelation of spontaneous firing lacks a distinct peak within 50 ms. **f2** Spontaneous firing shows phase preference close to LFP theta cycle peak. *Top* band-pass filtered LFP (3–6 Hz) and axo-axonic cell spikes (band-pass filtered at 0.3–5 kHz). *Bottom* histogram shows the firing phase preference (mean ± cSD) during LFP theta (18° bins, black line sine wave). **g** Molecular analysis and synaptic targets identify the axo-axonic cell. *Left* the neurobiotin-filled soma (NB, Alexa488, *white arrow*) is immunopositive for PV (Cy5) and immunonegative for the transcription factor SATB1 (Cy3: *asterisk*, nucleus of another PV+ cell). *Scale* 20 µm. *Right* electron micrograph of an axon initial segment (AIS) recognized by the membrane undercoating (*arrowheads*). It is innervated (*arrows*) by a neurobiotin-labelled bouton (*left*) of the axo-axonic cell visualized by electron opaque peroxidase reaction end-product. *Scale* 0.5 µm. **h** Synaptically-evoked average Pr of the axo-axonic cell in baseline and after TBS (*red*). Spontaneous firing (*black*) transiently suppressed from baseline only at 30 min post-TBS period (*P* < 0.005, ANOVA with Bonferroni test)

Fast-spiking interneurons defined by spike duration (1.02 ± 0.05 ms, *n* = 7 cells) (Fig. 3a). On average exhibited higher spontaneous discharge levels than pyramidal cells (Table 3) (Klausberger et al. 2003). Autocorrelogram of spontaneous firing exhibited no peaks at intervals up to 50 ms indicating a lack of complex spikes in the cells. Anatomical analysis revealed three of the fast-spiking cells as basket cells (Fig. 3), one axo-axonic cell (AAC) and one bistratified cell (Fig. 4) (Klausberger et al. 2003; Klausberger and Somogyi 2008). All five recovered cells were immunopositive for PV. In line with previous findings, the AAC was immunonegative for the transcription factor SATB1 (Viney et al. 2013) (Table 3). The axon of the AAC was weakly labelled by neurobiotin and appeared in both the pyramidal cell layer and in *stratum oriens*. We tested some boutons by electron microscopy and they were aligned with axonal initial segments (Fig. 4). The preferred firing phase of one basket cell (Table 3) was on the early descending slope of the LFP theta cycle close to where axo-axonic cells may also fire (Klausberger et al. 2003). Therefore, we tested some of the axon terminals for postsynaptic targets by electron microscopy and found that they innervated cell bodies and dendrites (not shown) consistent with this neuron being a basket cell. The bistratified cell showed characteristic axon distribution in *strata radiatum, pyramidale* and *oriens* (Halasy et al. 1996). Its dendrites did not extend into *stratum lacunosum*-*moleculare*. The cell was immunopositive for NPY (Klausberger et al. 2004). Two fast-spiking cells were not recovered following the labeling attempt, but both cells had preferred spontaneous firing close to the LFP theta cycle trough indicating possible dendrite-targeting PV+ cells (Klausberger et al. 2003, 2004). Spiking features of the cells including their spontaneous firing during LFP theta periods in baseline are detailed in Table 3.

**Table 3:** Summary of spontaneous firing properties and spike kinetics of fast-spiking interneurons

Spontaneous spiking properties					Immunohistochemistry
Cell code, cell type	Firing rate during LFP theta (Hz)	Mean angle phase (±cSD) to LFP theta cycle (°)	Depth of modulation (*r*) during the theta and *n* of cycles	Spike duration (ms) (mean ± SEM)	
PL210213, BC	21.6	239 ± 71	0.24, *n* = 1804	1.07 ± 0.01	PV+
PL230313, BC	14.9	305 ± 75	0.14, *n* = 4947	0.88 ± 0.03	PV+, CB1R−
PL190912, BC	20.1	303 ± 64	0.38, *n* = 1842	1.11 ± 0.02	PV+
PL311012, AAC	35.4	213 ± 65	0.36, *n* = 825	0.96 ± 0.02	PV+, SATB1−
PL200711, Bistratified	2.25	358 ± 48	0.64, *n* = 243	1.08 ± 0.03	PV+, NPY+
PL020213, Unidentified	14.0	311 ± 67	0.31, *n* = 7114	0.83 ± 0.06	N/A
PL030412, Unidentified	13.2	337 ± 75	0.13, *n* = 18579	1.17 ± 0.03	N/A

Spontaneous firing rates of the recorded fast-spiking interneurons during LFP theta oscillations and their average firing phase during LFP theta cycles (cSD indicates circular standard deviation). The axo-axonic cell showed firing preference close to the peak of theta cycles followed by basket cells firing along the descending slope. A bistratified cell and the two unrecovered fast-spiking cells fired close to the trough. All cells showed fast spike kinetics (spike duration below or close to 1 ms). Immunohistochemical reactions

+, positive; −, negative; N/A, not applicable

In baseline conditions, afferent stimulation with estimated half-maximal intensity elicited spikes with 0.59 ± 0.14 probability and 9.71 ± 0.62 ms latency (*n* = 7) (Table 4). Unlike in the pyramidal cell recordings, detectable fEPSP was generated in only one of the seven recordings with the stimulus intensity criteria used (Buzsaki and Eidelberg 1982b). Following TBS, at >15 min, two basket cells exhibited long-lasting depression of the evoked spike probability from 0.52 to 0.35 (*P* < 0.005) and from 0.32 to 0.19 (*P* < 0.01). One basket cell showed LTP-like potentiation (*P* < 0.05) (Chi square test) (Fig. 3c–e) (Peterfi et al. 2012). In the case of the basket cell with LTP, during the one second prior to TBS, the LFP was dominated by slow oscillations (1–3 Hz) in contrast to the two basket cell recordings with LTD, which were dominated by 3–6 Hz oscillations in the LFP. The axo-axonic cell did not show significant long-lasting change in the probability or delay of evoked spikes (Chi square test) although its spontaneous firing level was reduced long term (>15 min post-TBS, see Table 4) (Nissen et al. 2010) and the LFP during the one second immediately prior to TBS was dominated by 3–6 Hz oscillations. In contrast, the identified bistratified cell and the two unidentified fast-spiking cells showed LTP of the stimulus-evoked spike probability after TBS (Fig. 4c–h; Table 4). In the cells with LTP, spontaneous firing level changed long term (post-TBS >15 min) only in one unidentified cell (PL020213) showing a moderate decrease (see Table 4). The TBS was applied at a time dominated by 3–6 Hz oscillations in the LFP for one unidentified fast-spiking cell with LTP (PL020213), and by slow oscillations (1–3 Hz) in the other two experiments resulting in LTP (PL030412, PL200711). LFP oscillation analysis results for all cells in this study are summarized in Table 7.

**Table 4:** Summary of firing of PV+ interneurons

Synaptically evoked spike properties and spontaneous firing rate during plasticity experiments									
Cell code and type	Probability to the afferent stimulation			Latency to the afferent stimulation (ms) (mean ± SEM)			Overall spontaneous firing of the cell during the experiment (Hz)		
	Base-line	Post-TBS (0–15 min)	Post-TBS (>15 min)	Baseline	Post-TBS (0–15 min)	Post-TBS (>15 min)	Base-line	Post-TBS (0–15 min)	Post-TBS (>15 min)
PL210213, BC	0.73	0.90***	0.86*	9.05 ± 0.07	9.22 ± 0.05	8.79 ± 0.09	18.0	11.5**	15.2
PL230313, BC	0.52	0.39*	0.35***	9.11 ± 0.46	9.34 ± 0.33	8.14 ± 0.38	15.9	14.3	15.1
PL190912, BC	0.32	0.19**	0.19**	7.04 ± 0.82	7.00 ± 0.70	7.36 ± 0.58	19.8	13.9**	12.9**
PL311012, AAC	0.69	0.70	0.75	12.04 ± 0.28	11.12 ± 0.29	11.78 ± 0.35	21.1	21.2	14.7**
PL200711, Bistr.	0.61	0.69*	0.80***	11.40 ± 0.16	10.49 ± 0.12	10.08 ± 0.15	0.6	0.6	0.5
PL020213, Unid	0.60	0.87***	0.81***	9.38 ± 0.24	9.45 ± 0.25	9.19 ± 0.25	12.8	11.3	9.4*
PL030412, Unid	0.69	0.93***	0.93***	9.94 ± 0.46	9.95 ± 0.35	9.89 ± 0.12	12.7	14.7*	12.7

Average probability (Chi square test) and latency (ANOVA with Bonferroni test) of the synaptically evoked spikes are compared during baseline and following TBS (post-TBS). Columns at right show the overall spontaneous firing frequency in the baseline and at early (0–15 min) and late (from 15 min until the end of recording) post-TBS time windows including theta and non-theta periods

* *P* < 0.05, ** *P* < 0.01, *** *P* < 0.005

The changes in spike probability in the fast-spiking cells were not associated with significant long-term alterations in the average spike delay or the spike delay variance. In the four cells with significant LTP (see Table 4) average spike probability increased from 0.66 ± 0.03 to 0.85 ± 0.03 (15–45 min post-TBS, *n* = 4, *P* < 0.01, Chi square test) with no change in the average spike delay (9.93 ± 0.51 vs. 9.49 ± 0.30 ms, *t* test). In parallel coefficient of variance (CV) of the delay time decreased from baseline in three of the four cells (from 0.13 to 0.08; from 0.08 to 0.05; from 0.28 to 0.16), but remained unaltered in one neuron (0.20 vs. 0.21). However, 1/CV^2^ of the four cells was not significantly changed after LTP (15–45 min) from baseline (*n* = 4, *t* test). Baseline-normalized 1/CV^2^ of the cells was 2.29 ± 0.47 (*n* = 4). Likewise, the spike average delay for the two basket cells with LTD was unaltered between the baseline and the depression (see Table 4). Spontaneous firing level during the recording, and the results of immunohistochemical reactions are shown in Table 3. Details on the evoked spike probability and delay are seen in Table 4.

### Plasticity of synaptic excitation in NOS+ ivy cells

**Fig. 5:**
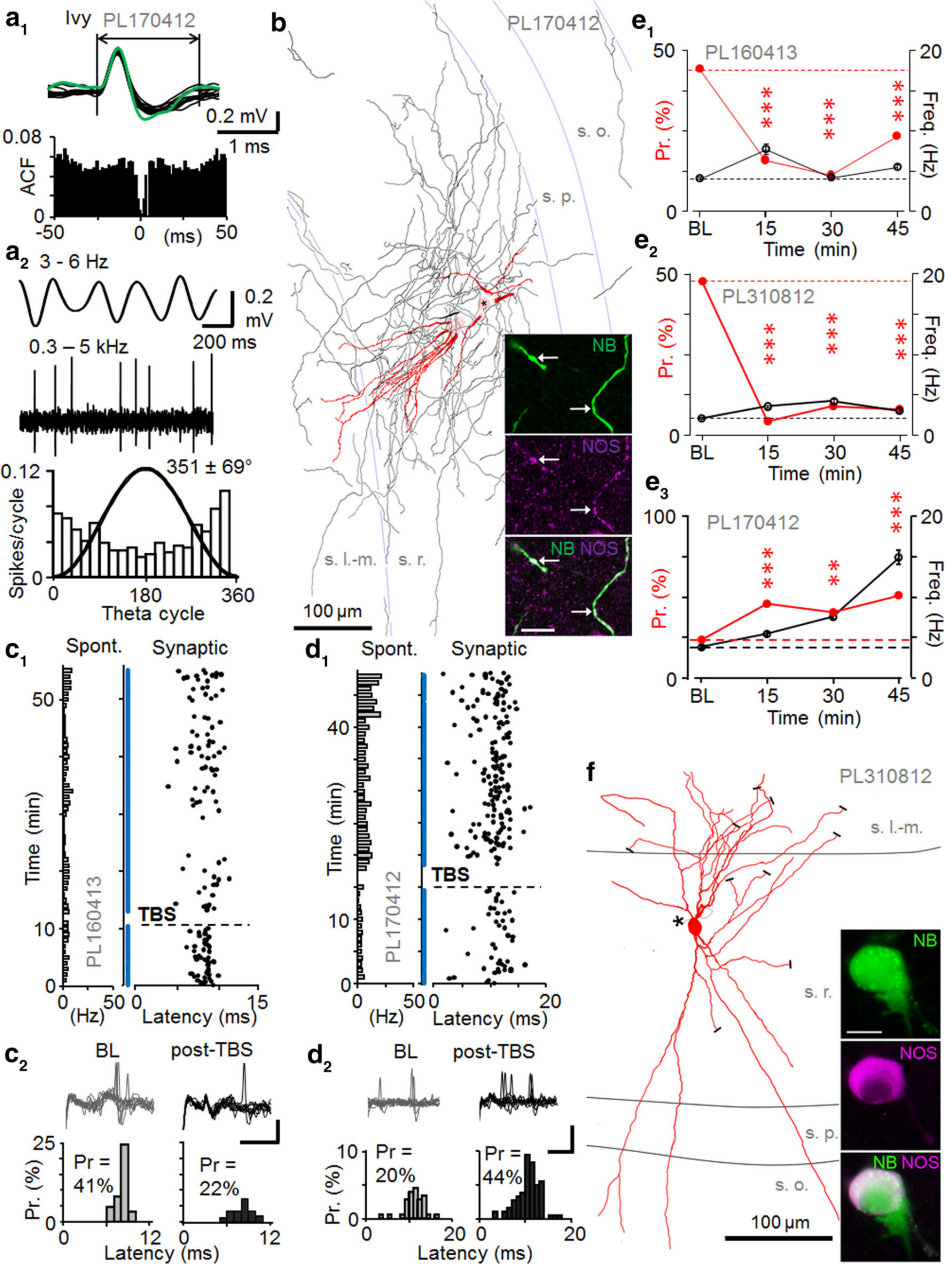
Persistent potentiation or depression of synaptic excitation in NOS+ ivy cells. **a** Spontaneous firing of an ivy cell (PL170412). **a1** *Top* superimposed spontaneous (*black*) and synaptically-evoked (*green*) spikes in the cell (spike waveform >1.5 ms). *Bottom* autocorrelogram of spontaneous firing. **a2** Spiking during LFP theta frequency (3–6 Hz) oscillations. *Top* band-pass filtered LFP (3–6 Hz) and ivy cell spikes (band-pass filtered at 0.3–5 kHz). *Bottom* spike histogram (18° bins) shows slight firing preference at the LFP trough (*black line* sine wave). **b** Partial reconstruction of the dendrites (*red*) and axon (*black* both from four 70 µm-thick sections) of the ivy cell with cell body in *stratum radiatum* (s.r.). *White matter* is to the *right*. Damage to the soma by the labelling resulted in a spillage of neurobiotin, but all the dendrites converged to one site (*circle*, *star*). The axon was most dense in *s.r*, but collaterals were present in all layers. Confocal microscopic images show NOS immunopostive (*purple*) neurobiotin-labeled (NB, *green*) dendrites. *Scale* 10 µm.** c**–**d** Ivy cells with LTD-like depression or LTP-like potentiation of action potential probability. **c** Persistent depression of spike probability by TBS (*horizontal line*) with no significant change in spike latency. **c1** Stimulation cycles with failures to evoke spike are shown by *blue dots*. *Horizontal bars on left* indicate spontaneous firing of the cell (1 min bins). **c2** Synaptically-evoked spikes in baseline and following TBS (30 min). *Scales* 0.2 mV, 3 ms. *Top* superimposed traces. *Bottom* histograms of spike probability (Pr) and delay (latency). Pr decreased from baseline (*P* < 0.005, Chi square test). **d** Another ivy cell showing persistent potentiation. **d1** Synaptically-evoked spike probability and delay (*black*) with failures (*blue*), and spontaneous firing on the *left*. **d2** *Top* traces in baseline and post-TBS. *Scales* 0.5 mV, 10 ms. *Bottom* histograms of the evoked spike occurrence. Probability (Pr) increased after TBS (*P* < 0.01, Chi square test). **e** Synaptic spike probability (*red*) at different times in three recorded and identified NOS+ ivy cells (**e1**–**e3**). Average spontaneous firing in parallel shown in *black*. Changes in Pr are compared to baseline (Chi square test). Spontaneous firing level was significantly changed long term from baseline in PL170412 (at 30 and 45 min *P* < 0.005, ANOVA with Bonferroni test). **f** Reconstruction of the ivy cell PL310812 with soma in *stratum radiatum* that showed LTD of spike probability (see **e2**). Dendrites (*red*) are shown from three 70 μm thick sections, cut dendrites indicated with *black bars*, and *asterisk* shows the origin of the axon (*black*). *Inset* the neurobiotin (NB, *green*) labelled soma is immunopositive for NOS (*purple*); a microglial cell attached at lower right took up neurobiotin. *Scale* 10 μm

Identified ivy cells (Fuentealba et al. 2008) showed slow spike kinetics (1.60 ± 0.09 ms, *n* = 3) close to that found in pyramidal cells (Fig. 5a). Anatomical analysis of the recorded cells uncovered characteristic dense axons in *stratum radiatum* and immunopositivity for neuronal nitric oxide synthase (NOS) (Fuentealba et al. 2008; Szabo et al. 2012; Somogyi et al. 2012; Armstrong et al. 2012) (Fig. 5b). The cell bodies were at different laminar locations: PL170412 was in *stratum radiatum* (Fig. 5b), PL310812 in *stratum radiatum* one third from *the border with stratum lacunosum*-*moleculare* (Fig. 5f)*, and PL160413 was located in s*. pyramidale. The dendritic trees were also variable. The ivy cells fired with highest probability after or close to the LFP theta cycle trough (Table 5). Their average spontaneous firing rate during theta frequency (3–6 Hz) oscillations was similar to that of pyramidal cells (Table 5).

**Table 5:** Spontaneous firing properties and spike kinetics of ivy cells

Spontaneous spiking properties					Immunohistochemistry
Cell code, cell type	Firing rate during LFP theta (Hz)	Mean angle phase (±cSD) to LFP theta cycle (°)	Depth of modulation (*r*) during the theta and *n* of cycles	Spike duration (ms) (mean ± SEM)	
PL170412, Ivy	4.0	352 ± 69	0.28, *n* = 388	1.49 ± 0.02	NOS+
PL310812, Ivy	1.5	34 ± 66	0.33, *n* = 346	1.88 ± 0.02	NOS+, proCCK−
PL160413, Ivy	4.5	99 ± 58	0.48, *n* = 2332	1.54 ± 0.04	NOS+, PV−, proCCK−

Firing phase preference during LFP theta cycles was calculated from spontaneous activity during theta epochs (cSD means circular standard deviation). All three cells had low spontaneous firing rates. The mean firing phase of two ivy cells was on the early ascending slope of theta cycles and the third ivy cell coupled its spikes to the theta cycle troughs. Action potentials showed slow kinetics (≥1.5 ms duration). Immunohistochemical reactions; + positive, − negative

During baseline conditions, single shock stimulation evoked a spike with 0.39 ± 0.07 probability and 9.63 ± 0.91 ms delay to the stimulation (*n* = 3) without detectable fEPSP in any of the recordings. Two ivy cells showed long-lasting depression of spike probability at >15 min following TBS (for each cell *P* < 0.005, Chi square test). One ivy cell exhibited long-lasting potentiation of spike probability from 0.24 during baseline to 0.52 post-TBS (*P* < 0.005, Chi square test) (Fig. 5c–e). None of the three cells showed significant change in average spike delay (*t* test) (see Table 6). Interestingly, both ivy cells with LTD also showed increase in spike delay time coefficient of variance (CV) from baseline after TBS (>15 min) (from 0.11 to 0.29 and 0.10 to 0.21), whereas the cell with potentiation showed no alteration (0.23 vs. 0.25).

**Table 6:** Summary of firing of ivy cells

Synaptically evoked spike properties and spontaneous firing rate during plasticity experiments									
Cell code and type	Probability to the afferent stimulation			Latency to the afferent stimulation (ms) (mean ± SEM)			Overall spontaneous firing of the cell during the experiment (Hz)		
	Base-line	Post-TBS (0–15 min)	Post-TBS (>15 min)	Baseline	Post-TBS (0–15 min)	Post-TBS (>15 min)	Base-line	Post-TBS (0–15 min)	Post-TBS (>15 min)
PL170412, Ivy	0.24	0.47***	0.52***	11.30 ± 0.47	11.29 ± 0.28	10.12 ± 0.47	3.8	5.5	15.0***
PL310812, Ivy	0.48	0.03***	0.06***	9.43 ± 0.22	9.84 ± 1.63	11.23 ± 1.50*	2.2	3.3	3.6*
PL160413, Ivy	0.44	0.16***	0.23***	8.16 ± 0.13	8.77 ± 0.53	8.05 ± 0.31	4.1	7.7	5.5*

Table shows comparisons of the average probability (Chi square test) and latency (ANOVA with Bonferroni test) of the synaptically evoked postsynaptic spikes during baseline and following high-frequency theta-burst stimulation (post-TBS). Columns at right show the overall spontaneous firing frequency in the baseline and at early (0–15 min) and late (from 15 min until the end of recording) post-TBS time

* *P* < 0.05, *** *P* < 0.005

The spontaneous firing level of all three cells increased long-term (>15 min post-TBS) from baseline (*P* < 0.05 and *P* < 0.005, ANOVA with Bonferroni test) (Table 6). All three recordings from ivy cells showed theta-like (3–6 Hz) activity in the LFP during the period one second prior to TBS (see Table 7). Moreover, the LFP power spectrogram for the ivy cell PL170412 also showed an additional clear peak in the slow frequency range (1–3 Hz) (data not shown). In this neuron TBS led to LTP in contrast to the other two cells. Moreover, there was a quarter of oscillatory cycle difference in the preferential theta phase of firing of this neuron compared to the other two cells (see Table 5).

**Table 7:** Summary of LFP index pre-TBS (1 s) and post-TBS (1 s) and long-term plasticity changes of the recorded neurons

Cell ID	Cell type	Cell code	Pre-TBS	Post-TBS	Outcome
PL120811	PC	PL120811	−0.1	−0.5	LTP
PL240113	PC	PL240113	0.1	−0.3	LTP
PL260313	PC	PL260313	−0.5	−0.7	LTP
PL210213	BC	PL210213	−0.2	−0.5	LTP
PL230313	BC	PL230313	0.2	−0.1	LTD
PL190912	BC	PL190912	0.3	0.3	LTD
PL311012	AAC	PL311012	0.4	0.2	No change
PL200711	Bistratified	PL200711	−0.9	−0.6	LTP
PL020213	Unidentified	PL020213	0.8	0.8	LTP
PL030412	Unidentified	PL030412	−0.6	−0.8	LTP
PL170412	Ivy	PL170412	0.4	0.5	LTP
PL310812	Ivy	PL310812	0.4	0.4	LTD
PL160413	Ivy	PL160413	0.7	0.3	LTD

Wavelet power spectrogram of the LFP was calculated in time windows before and after TBS. Index values of 1 and −1 would represent spectral power components only in the frequency ranges of 3–6 or 1–3 Hz, respectively; 0, represents exactly same average power in both frequency ranges. The average wavelet power across a range of frequencies was determined as the weighted sum of the wavelet power spectrum over the respective frequencies. In the group of fast-spiking cells, TBS only changed the LFP power ratio in one cell (PV+ basket cell recording PL230313)

### Analysis of spike delay time in all cells with LTP or LTD

In each identified cell population, the pyramidal cells, the fast-spiking interneurons and the ivy cells, individual neurons showing LTP or LTD comprise a small sample making statistical analyses of the spike delay properties unreliable or untestable. Therefore, we analyzed the spike probability and delay properties pooling all cells showing either LTP or LTD. In the cells showing LTP, spike probability was potentiated from 0.50 ± 0.06 to 0.84 ± 0.05 at 15–45 min post-TBS (*n* = 8, *P* < 0.01, *t* test). Although the average spike delay, measured in the same time windows remained unchanged (10.64 ± 0.43 vs. 10.24 ± 0.41 ms), the spike delay time coefficient of variance showed a significant reduction from baseline (compared as 1/CV^2^, *n* = 8, *P* < 0.05, *t* test) with baseline-normalized 1/CV^2^ of 1.96 ± 0.35 (*n* = 8). In cells showing LTD, spike probability decreased from 0.44 ± 0.04 in baseline to 0.21 ± 0.06 at 15–45 min post-TBS, *n* = 4, *P* < 0.05, *t* test), but no significant change was detected in either the average delay (8.43 ± 0.54 vs. 8.70 ± 0.86 ms, *n* = 4, *t* test) or the 1/CV^2^ (baseline-normalized 0.65 ± 0.28) (*n* = 4, *t* test).

### Comparison of postsynaptic cell firing and network oscillation activity with the plasticity in fast-spiking cells

**Fig. 6:**
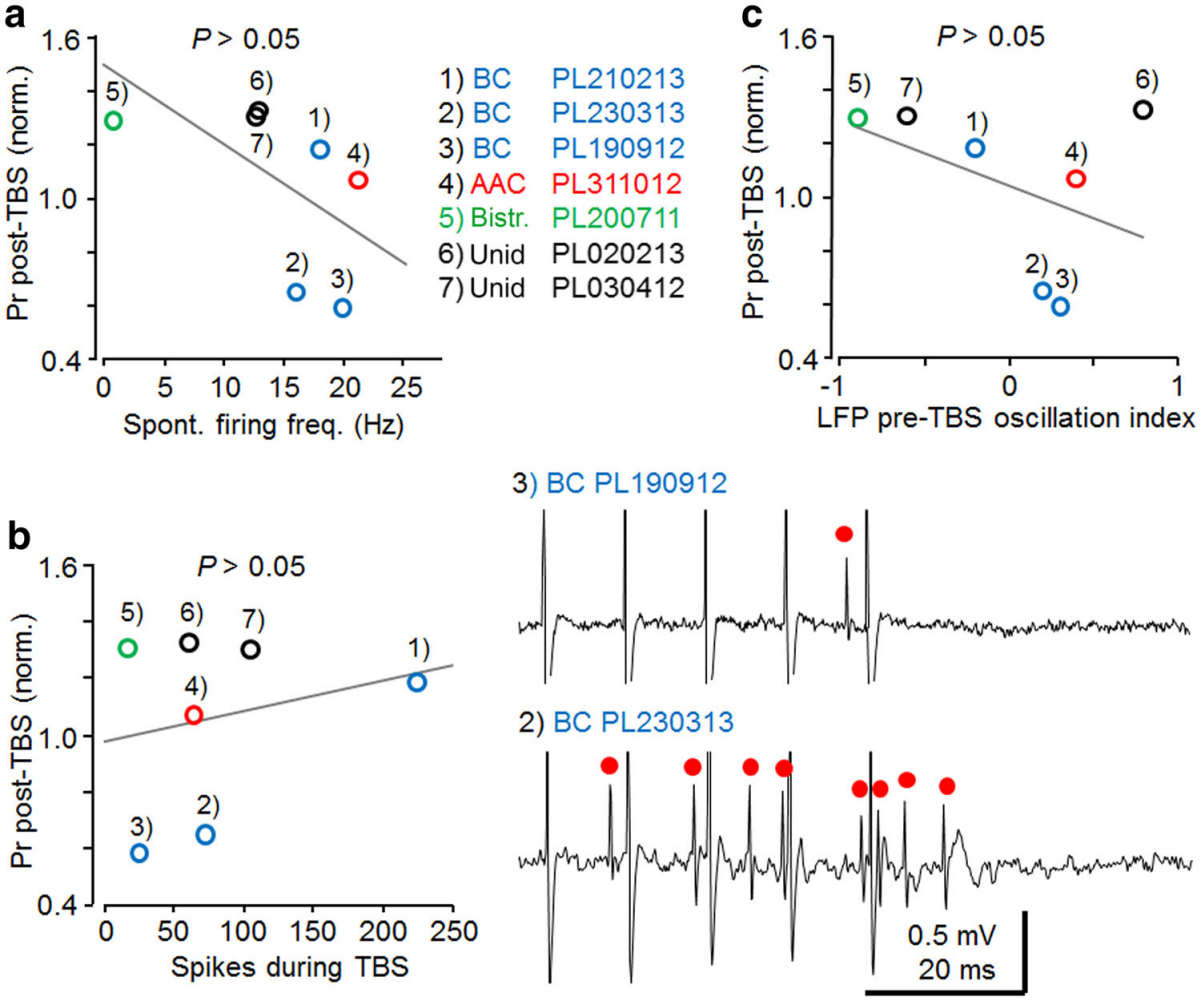
The effect of postsynaptic cell firing and hippocampal network oscillatory states on plasticity in fast-spiking interneurons. Individual cells are annotated and cell types shown in different colors as indicated. **a** Spontaneous firing level of the recorded fast-spiking interneurons and baseline-normalized plasticity (>15 min post-TBS) failed to show significant correlation (*n* = 7, Pearson’s test). The correlation coefficient suggests linear relationship between the variables (*r* ^*2*^ = 0.36, *n* = 7, Pearson’s test), but correlation is not significant (*P* > 0.05). **b** The firing level of the cells during afferent TBS, on average, did not explain whether LTP or LTD was generated in the fast-spiking interneurons. However, note differences in the direction of plasticity for PV+ basket cells with mean rate >200 Hz vs. mean rate <100 Hz. *Left* the plot shows averaged baseline-normalized spike probability (Pr) post-TBS and the number of spikes generated during TBS (*r* ^2^ = 0.05). No correlation was found between the factors (*n* = 7, Pearson’s test). *Right* sample traces (one theta burst of 5 pulses at 100 Hz) in the two LTD-exhibiting PV+ basket cells with very different TBS-associated firing. *Red dots* mark evoked action potentials. **c** Relationship of the LFP index for pre-TBS, based on wavelet power spectrogram (1 s before TBS), and long-term plasticity of evoked spike probability (Pr) in the fast-spiking interneurons. Index values of 1 and −1 represent spectral power components only in the frequency ranges of 3–6 or 1–3 Hz, respectively; 0, represents equal average power in both frequency ranges. As a group, the fast-spiking cells did not show correlation between pre-TBS LFP oscillatory components and the direction of plasticity (*n* = 7, Pearson’s test) (*r* ^2^ = 0.19)

We studied whether differences in firing rates of the fast-spiking interneurons, or any potential change in underlying LFP activity due to TBS could explain differences observed in the plasticity results. For the analyses, synaptically-evoked spike probability following TBS (post-TBS >15 min) was normalized by baseline in each cell. We found that although the correlation coefficient might indicate a linear relationship between the spontaneous firing level of fast-spiking interneurons and the baseline-normalized plasticity of spike probability (*r* ^*2*^ = 0.36, *n* = 7, Pearson’s test), correlation of the variables was not significant (*P* = 0.15) and cells showing either LTD or LTP had similar spontaneous firing rates (Fig. 6a). In addition, the firing level of fast-spiking cells during the TBS did not correlate with the plasticity generated (*r* ^*2*^ = 0.05, *n* = 7, Pearson’s test). However, mean firing >200 Hz during TBS in one PV+ basket cell was accompanied by LTP, contrary to LTD induction in the other two PV+ basket cells with firing rates <100 Hz during TBS (Fig. 6b). Yet, similar firing levels of <100 Hz during TBS observed for the other fast-spiking cells elicited LTP (Fig. 6b). Interestingly, when we compared baseline-normalized plasticity with LFP power ratio of two spectral components (1–3 and 3–6 Hz) measured in 1 s immediately prior to the TBS (see “Methods”), we found that LTD in the two basket cells and in the two ivy cells was evoked from a predominant 3–6 Hz LFP oscillatory network state without clear 1–3 Hz component (Table 7). On the contrary, LTP was evoked in the basket cell when 1–3 Hz oscillatory component dominated the LFP 1 s before TBS, and the hippocampus showed both 1–3 and 3–6 Hz LFP oscillatory activity in the 1 s before TBS for the ivy cell (PL170412) with LTP. However, in the two unidentified fast-spiking cells LTP was observed with either 1–3 or 3–6 Hz predominant LFP component, and TBS to the axo-axonic cell that showed no lasting plasticity had predominant 3–6 Hz LFP oscillation before the TBS. Therefore, as a group, the fast-spiking cells did not show correlation between plasticity and LFP oscillatory patterns (*r* ^*2*^ = 0.11, *n* = 7, Pearson’s test) immediately before the TBS (Fig. 6c). Due to the small number of identified cells of the same type, we cannot exclude that the different outcome in individual basket and ivy cells were a result of the difference in oscillatory network states, as reflected in the LFP, at the time of initiating the TBS.

With the exception of the PV+ basket cell recording PL230313, TBS only slightly changed the LFP power ratio of the two spectral components (1–3 and 3–6 Hz) in 1 s following TBS as compared to 1 s prior TBS (Table 7). The results suggest that, on average, the firing levels of the interneurons or the potential change in network excitability evoked by TBS in the experiments were unlikely causes of the potentiation or the depression generated by TBS.

## Discussion

Our experiments demonstrate that extracellullar recording with juxtacellular labeling for neuron identification allows a stable and non-invasive approach for measurements of changes in synaptic excitation in hippocampal neurons in vivo. We have shown activity-induced long-term plasticity of evoked spike probability in identified hippocampal interneuron types in the CA1 area. In line with previous reports from ex vivo slice preparations we demonstrated that PV+ basket cells can generate either LTP or LTD following high frequency afferent stimulation (Nissen et al. 2010; Peterfi et al. 2012; Le Roux et al. 2013; Campanac et al. 2013; Camire and Topolnik 2014). However, we found that NOS+ ivy cells in the CA1 area can show either potentiation akin to LTP or LTD-like depression in vivo, which differs from an earlier report using slices, in which only LTP has been reported (Szabo et al. 2012). Interestingly, a dendrite-targeting bistratified cell and one putative but unidentified dendrite-targeting fast-spiking cell showed LTP in vivo. Both LTP and LTD have been reported in dendrite-targeting CA1 PV+ interneurons (Nissen et al. 2010; Oren et al. 2009; Perez et al. 2001). The occasional failure of some PV+ cells to show long-term synaptic plasticity, as seen here in one AAC, has been reported in slices (Nissen et al. 2010). All the three pyramidal cells showed robust LTP as has been demonstrated both in vitro and in vivo. Our results show that distinct CA1 interneuron types exhibit long-term plasticity in vivo and PV+ basket and NOS+ ivy cells can show either LTP or LTD.

A possible explanation to the variable plasticity results in basket cells and ivy cells may be a different activation of receptors and molecular pathways by electrical stimulation. Although CA1 interneurons mostly show NMDAR-independent potentiation and depression of synaptic excitation (Nissen et al. 2010; Campanac et al. 2013; Szabo et al. 2012; Peterfi et al. 2012), PV+ basket cells also exhibit NMDAR-dependent LTP in some of their excitatory afferents (Le Roux et al. 2013). Ketamine, which was used here as a supplementary anesthetic inhibits NMDARs (Harrison and Simmonds 1985), and may have differentially inhibited NMDAR-mediated plasticity in some of the experiments. Hence, different contribution of NMDAR-mediated LTP (Le Roux et al. 2013) might explain some of the variability in the plasticity results observed in the PV+ basket cells in our study.

A second possible explanation of variability is differences in neuromodulator release in the experiments and their effect on interneuron plasticity. Endocannabinoids are powerful modulators of long-term plasticity in glutamatergic synapses onto PV+ neurons in the CA1 area. Experiments in slices have demonstrated that LTD rather than LTP is induced in these cells when endocannabinoids are released by high-frequency stimulation (Peterfi et al. 2012; Le Roux et al. 2013; Nissen et al. 2010; Campanac et al. 2013). Importantly, activation of endocannabinoid system results in LTD not only in PV+ interneurons, but also in many non-fast spiking GABAergic neurons (Edwards et al. 2012). It is possible that whether LTP or LTD is generated in NOS+ ivy cells also depends on concomitant endocannabinoid release or is due to the effects of other neuromodulators. This could also explain some discrepancies reported in long-term plasticity between ex vivo and in vivo conditions in these interneuron types; intact brain circuits in vivo might provide stronger endocannabinoid release and cannabinoid receptor activation than the conditions in brain slices. Likewise, antidromic activation of fibers innervating hippocampus from extrahippocampal loci could contribute to the plasticity. Activation of acetylcholine receptors has been demonstrated to promote NMDAR-independent LTP in CA1 area interneurons through both nicotinic and muscarinic receptors (Le Duigou et al. 2015; Griguoli et al. 2013). However, the impact of many other neuromodulatory signaling pathways on interneuron long-term plasticity is unknown. Activation of serotonergic, noradrenergic or dopaminergic fibers antidromically in the hippocampus either by electrical stimulation or occurring naturally in behaving animal have strong acute effects on GABAergic interneuron function (Bohm et al. 2015; Rosen et al. 2015; Maccaferri 2011). Dopaminergic fiber activity facilitates LTP in CA1 pyramidal cells and promotes hippocampal spatial memory persistence (Li et al. 2003; McNamara et al. 2014), but any impact on hippocampal interneuron plasticity remains to be tested.

Thirdly, we also analyzed the potential influence of rhythmic network states at the time of TBS application on plasticity in the interneurons. In general, the presence or absence of 1–3 and 3–6 Hz frequency oscillatory components in the LFP, did not explain whether LTP or LTD was generated in fast-spiking interneurons as a group. However, because of low number of cells the results on LFP oscillatory patterns and the direction of plasticity needs further investigation. We also observed that, on average neither the spontaneous firing rate of the neurons, nor their discharge during TBS correlated with LTP or LTD. We cannot exclude the possibility that in some cases altered excitability during the course of the experiment had an impact on the synaptically-evoked spike probability. For instance, ivy cell PL170412 with LTP showed a prominent increase in spontaneous firing after the TBS which might reflect changes in its excitability. However, in most cells the spontaneous firing level changes were small or moderate and in many cells were of opposite direction than the evoked spike probability.

Finally, it is important to consider if the stimulus-evoked spikes and their plasticity were mediated via a monosynaptic excitatory pathway to the recorded neurons or possibly by polysynaptic activity triggered in the antidromically stimulated CA3 area. We confirmed that stimulation from the contralateral left CA1 area elicited a phase-locked short-delay field EPSP in the CA1 of the right hemisphere (Bliss and Lomo 1973; Buzsaki and Eidelberg 1982b) in all experiments included in this study. In addition, the phase-locked action potentials in the recorded cells to the contralateral stimulation were generated with short delay and small jitter that correspond to signaling through a monosynaptic excitatory pathway (Buzsaki and Eidelberg 1982a, b). Neither the LTP-like spike probability potentiation nor the depression were associated with significant changes in the average spike delay in the reported cells (Maccaferri and McBain 1996; Buzsaki and Eidelberg 1982b), but in cells with LTP, the synaptically evoked spike delay variance decreased in parallel with the increased spike probability (Pouille and Scanziani 2001; Lamsa et al. 2005). Although we were unable to directly demonstrate monosynaptic generation of the spikes, which would have required intracellular recordings, the above lines of evidence indicate stimulus-evoked monosynaptic spike generation in these cells.

Although synaptic long-term plasticity provides a valid explanation to the potentiation and depression of the evoked spike probability reported here, other mechanisms may also be involved. High-frequency local stimulation can increase intrinsic excitability of some interneurons (Ross and Soltesz 2001; Campanac et al. 2013). In parallel with synaptic LTP, postsynaptic cell input resistance can increase through decreased K+ channel-mediated conductance in PV+ cells (Campanac et al. 2013). Similar increase in input resistance has been reported in dentate gyrus interneurons following local high-frequency stimulation (Ross and Soltesz 2001). Hence, potentiation of the synaptic excitation could be explained at least partly by changes in the postsynaptic cell intrinsic excitability and EPSP-spike coupling. In addition, compensation of synaptic LTD by increased intrinsic excitability may explain the lack of plasticity in the axo-axonic cell observed here. However, it is noteworthy that in hippocampal slice experiments the electrical stimulation is usually applied close to the recording site, and it is unclear to what extent stimulation in the close proximity contributes to the non-synaptic plasticity mechanisms. In our in vivo experiments the stimulation was applied far from the postsynaptic recording site in the contralateral hippocampus. However, we cannot exclude that in addition to synaptic plasticity non-synaptic mechanisms (Campanac et al. 2013) contributed to the spike probability changes reported here.

Spatial learning and memory storage in the hippocampus are associated with dynamic and persistent changes in communication between excitatory pyramidal place cells and their postsynaptic inhibitory interneurons in the CA1 area (Dupret et al. 2013). In a novel environment the establishment of new place cells is associated with long-lasting changes in their activation of fast-spiking CA1 interneurons. As a consequence, place cells can either increase or decrease their spike coupling to as yet unknown types of interneurons (Dupret et al. 2013). One interpretation for these results is that learning-associated neuronal activity generates long-term plasticity between the excitatory place cells and their postsynaptic interneurons in the CA1 area, as demonstrated here in vivo. If so, use-dependent LTP and LTD could contribute to learning processes in the hippocampus (Kullmann and Lamsa 2007), and might also be driven by synchronous high-frequency discharges in pathological conditions (Jefferys 2014). In line with results obtained in vitro (Kullmann and Lamsa 2011), the form of plasticity may depend on the identity of the interneurons and their synaptic properties. Both PV+ basket cells and NOS+ ivy cells were able to generate potentiation and depression, whereas other cells showed LTP in these conditions (Kullmann and Lamsa 2011).
